# Polydopamine-based loaded temozolomide nanoparticles conjugated by peptide-1 for glioblastoma chemotherapy and photothermal therapy

**DOI:** 10.3389/fphar.2023.1081612

**Published:** 2023-01-18

**Authors:** Hao Wu, Tianyi Zhang, Qi Liu, Min Wei, Yuping Li, Qiang Ma, Lianhui Wang, Yufu Zhu, Hengzhu Zhang

**Affiliations:** ^1^ Graduate School of Dalian Medical University, Dalian, China; ^2^ Department of Neurosurgery, Clinical Medical College, Yangzhou University, Yangzhou, China; ^3^ Nanjing University, Nanjing, China; ^4^ Department of Neurosurgery, The First Hospital of Yu Lin, Yulin, China; ^5^ Institute of Advanced Materials, Nanjing University of Posts and Telecommunications, Nanjing, China; ^6^ Department of Neurosurgery, The Affiliated Hospital of Xuzhou Medical University, Xuzhou, China

**Keywords:** temozolomide, polydopamine, PEP-1, glioblastoma, nanoparticles, chemo/photothermal therapy

## Abstract

**Purpose:** Nanoparticles (NPs) of the polydopamine (PDA)-based,loaded with temozolomide (TMZ) and conjugated with Pep-1 (Peptide-1) as a feasible nano-drug delivery system were constructed and utilized for chemotherapy (CT) and photothermal therapy (PTT) of glioblastoma (GBM).

**Method:** PDA NPs were synthesized from dopamine (DA) hydrochloride and reacted with TMZ to obtain the PDA-TMZ NPs and then the PDA NPs and the PDA-TMZ NPs were conjugated and modified by Pep-1 to obtain the Pep-1@PDA NPs and Pep-1@PDA-TMZ NPs *via* the Schiff base reaction (SBR), respectively.Their dimensions, charge, and shape were characterized by dynamic light scattering (DLS) and scanning electron microscope (SEM). The assembly of TMZ was verified by Fourier-transform infrared spectroscopy (FT-IR) and ultraviolet and visible spectroscopy (UV-Vis). The biostability of both the nanocarrier and the synthetic NPs were validated using water and fetal bovine serum (FBS). The antitumor activities of the PDA-TMZ NPs and Pep-1@PDA-TMZ NPs were verified in U87 cells and tumor-bearing nude mice.

**Results:** The prepared PDA NPs, PDA-TMZ NPs, Pep-1@PDA NPs, and Pep-1@PDA-TMZ NPs were regular and spherical, with dimension of approximately 122, 131, 136, and 140 nm, respectively. The synthetic nanoparticles possessed good dispersity, stability,solubility, and biocompatibility. No obvious toxic side effects were observed, and the loading rate of TMZ was approximately 50%.*In vitro* research indicated that the inhibition ratio of the Pep-1@PDA-TMZ NPs combined with 808 nm laser was approximately 94% for U87 cells and *in vivo* research was approximately 77.13%, which was higher than the ratio of the other groups (*p* < 0.05).

**Conclusion:** Pep-1 was conjugated and modified to PDA-TMZ NPs, which can serve as a new targeted drug nano-delivery system and can offer a CT and PTT integration therapy against GBM. Thus, Pep-1@PDA-TMZ NPs could be a feasible approach for efficient GBM therapy, and further provide some evidence and data for clinical transformation so that gradually conquer GBM.

## 1 Introduction

In clinical practice, GBM is the most common malignancy in the brain. GBM originates from glioma cells (Ostrom et al., 2020). Unfortunately, it is the survival rate of 5-years is only 23.5% (Ostrom et al., 2020). Currently, postoperative radiotherapy (RT) and CT are the conventional GBM treatment programs (Batash et al., 2017; Glaser et al., 2017). However, identifying which radiotherapy-based approach combined with chemotherapy are effective is a challenge due to GBM specificity, including diverse sites, histological types, and heterogeneity (Pearson and Regad, 2017). Postoperative CT, which can inhibit GBM recurrence, is often used to manage glioma, and these approaches directly affect the survival prognoses of the patients; TMZ, a clinical first-line chemotherapy drug for GBM treatment, has significant antitumor activity by inducing apoptosis to destroy glioma cells (Wang Y. et al., 2021). However, the dissolvability and stability of TMZ is relatively poorer in water and its half-life is only approximately 1.8 h. Moreover, TMZ can be instantly eliminated from *in vivo* circulation (Saenz del Burgo et al., 2014). TMZ also has some deficiencies, including inferior tumor targeting, considerable toxicities or adverse effects, drug resistance *via* DNA repair, and the regulation of several signaling pathways. The therapeutic effect of GBM remains unsatisfactory, even though timely operation and postoperative CT (Yu et al., 2022). GBM remains a challenging and valuable research topic. Basic researches and clinical trials exploring the RT and CT of postoperative GBM have paid closer and higher attention to the types of diverse nano-delivery system in recent years, because nano-delivery system can enhance the targeting ability of TMZ, even trigger and control the release of TMZ, and augment its biological half-life period to inhibit its degradation in the acidic environment of the tumor (Nie et al., 2019; Wang L. et al., 2021). The super-strong adhesion, better photothermal conversion and good biocompatibility of PDA, and its capacity to promote the molecular biology characteristics and upgrade bioavailability, supports the use of PDA with TMZ to improve delivery (Bi et al., 2018). Meanwhile, the PTT of PDA is beneficial for enhanced tumor therapy due to the synergistic effect, especially some multi-mode therapeutics such as the photodynamic therapy (PDT) combined with the PTT can convert molecular oxygen (O_2_) into cytotoxic reactive oxygen species (ROS) to destroy tumor cells. (Shao et al., 2020). More, Pep-1, a specific IL-13Ra2 ligand, was proved to improve the ability of traversing the blood brain barrier (BBB) and cytomembrane and targeted to GBM. Pep-1 can carry efficiently nanoparticles to traverse the BBB and enter into the nucleus (Wang et al., 2019).

**FIGURE 1:**
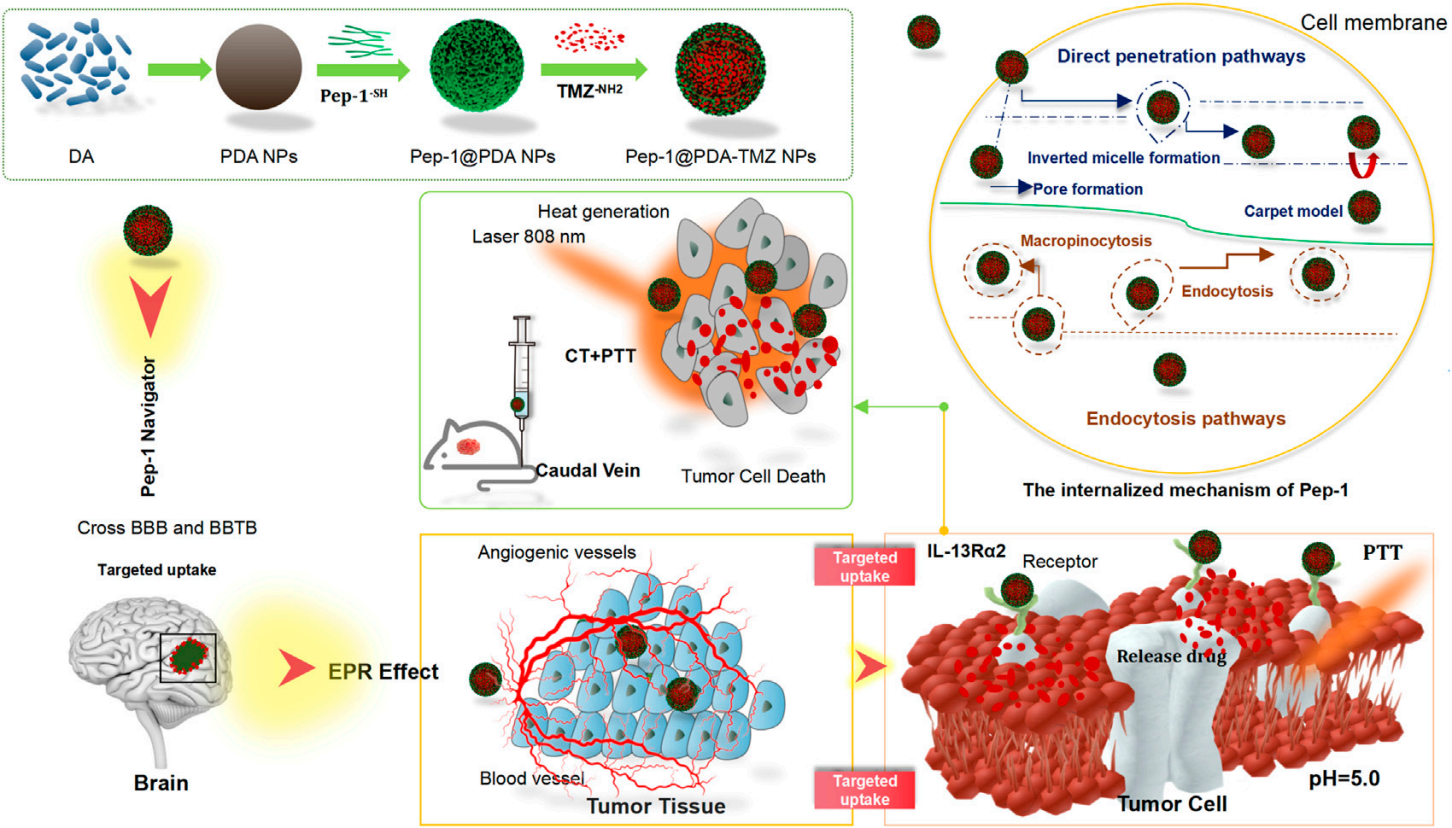
Graphical demonstration to display the synthesis of the Pep-1@PDA-TMZ NPs and the chemotherapy and photothermal therapy of Pep-1@PDA-TMZ NPs for glioblastoma.

**FIGURE 2:**
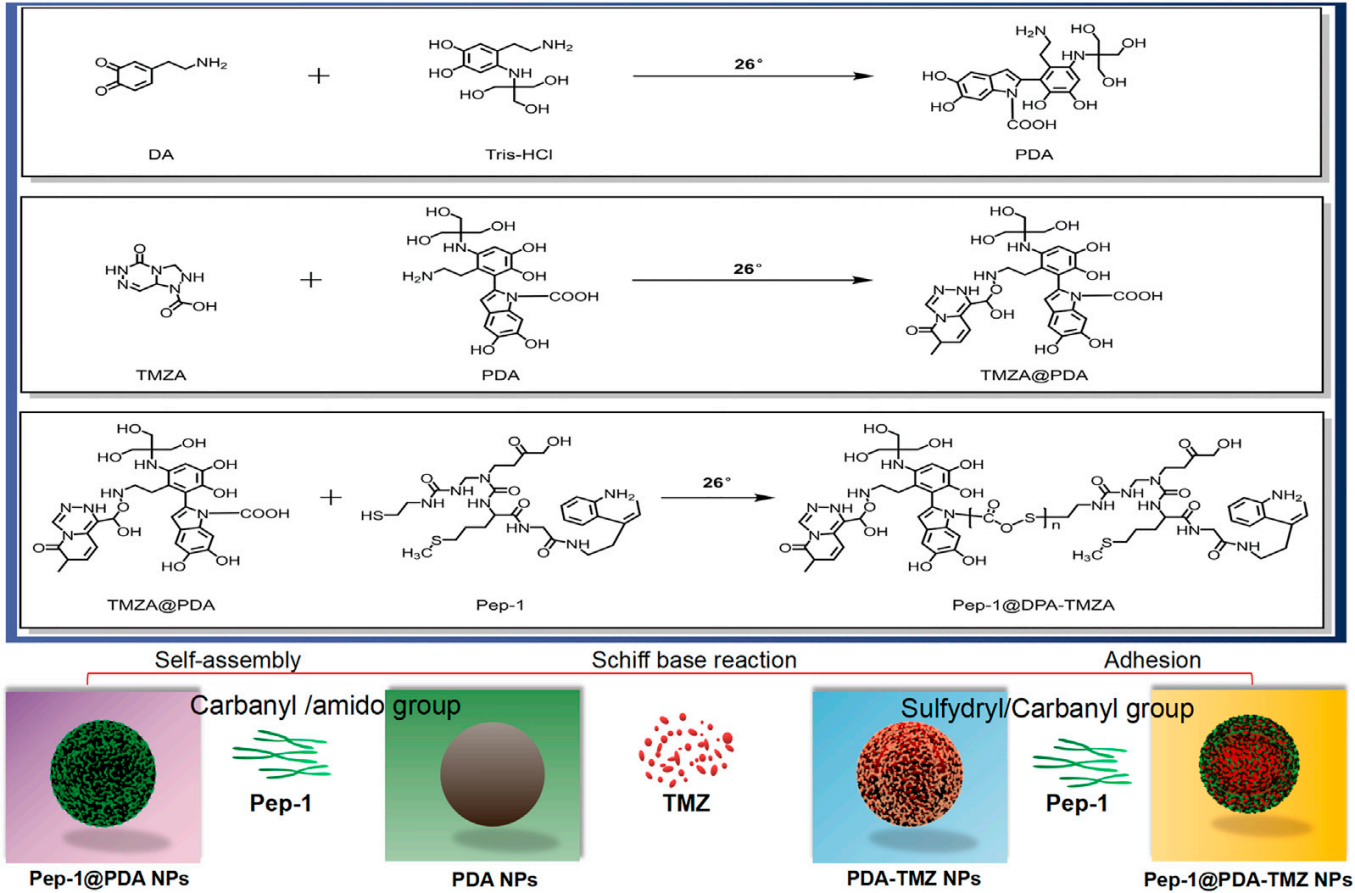
The features of the different NPs.

In this study, we designed a covalent self-assembly strategy by introducing dynamic Schiff base bonds (Sbb C=N) into the PDA NPs The formation of Sbb C=N occurred between the PDA amine group and the carbonyl groups of Pep-1 and TMZ *via* cross-linking during the SBR. Sbb C=N endowed the PDA-based NPs with pH-dependent degradation properties. After encapsulating TMZ, the versatile drug nano-delivery system Pep-1@PDA-TMZ NPs displayed that Pep-1 can be utilized as the promising accelerator entering tumor cells and the PDA NPs were the transport carrier; the system also demonstrated that pH-smart, responsive drug release is possible in the acidic tumor microenvironment (TEM) (Figure 1). The combination of carrier and drug can achieve the effects of synergistic antitumor by delivering CT and PTT. Pep-1@PDA-TMZ NPs with good biodegradability, pH-responsive drug release, and synergistic therapy are promising approach for optimizing the therapeutic efficacy of GBM (Zhang et al., 2015) (Figure 2).

## 2 Material and methods

### 2.1 Materials

Dopamine hydrochloride, Temozolomide,Temozolomide acid, Coumarin-6 and 2.5% Isoflurane were purchased from Sigma-Aldrich (St.Louis, United States). NH_2_-PEG-NH_2_ Amine, Tris-HCl,*N,N*-dimethylformamide (DMF), *N,N′*-dicyclohexylcar-bodiimide, 4-dimethylaminopyridine and Pep-1 were purchased from Nanjing Peptide Biotech Co., Ltd. All chemicals in cell experiments were purchased from Keygen Biotech Co., Ltd. (Nanjing, China).

### 2.2 Instruments and characterization

Ultraviolet and Visible Spectroscopy (UV-Vis) was conducted using a UV-3600 spectrophotometer (Shimadzu, Japan). Fourier-transform infrared spectroscopy (FT-IR) was performed and recorded on a Shimadzu IR Prestige-21 spectrometer by dispersing the sample in a KBr press. An EVO18 Scanning electron microscope (SEM) was utilized to obtain images, and the size of NPs was tested by using a Zeta PALS. The confocal laser scanning microscope (CLSM) images were attained using an FV1000 (Olympus, Japan). A Fotric 225 photothermal camera was utilized to record the temperature and capture images.

### 2.3 Cell lines and animals information

#### 2.3.1 Cell lines information

U87 cells were purchased from Nanjing Key gen Co., Ltd. The complete medium was 90% DMEM with 10% FBS; they were grown in an incubator at 37°C and 5% CO_2_ with saturated humidity. U87 cells were incubated for 24 h, followed by replacing the old culture with a medium containing different concentrations of the PDA NPs, PDA-TMZ NPs, Pep-1@PDA NPs, and Pep-1@PDA-TMZ NPs (200 μg/mL each) for a further 6 h incubation.

#### 2.3.2 Animal information

BALB/c nude mice were purchased from Shanghai Experimental Animal Co., Ltd. The Laboratory Animal Use License was SYXK (Su) 2017-0040. Mice were 4 weeks old and male, and five animals were assigned to each group. Eight groups were formed, requiring a total of 40 animals.

#### 2.3.3 Ethics statement

Studies involving animal participants were reviewed and approved by the Experimental Animal Welfare Ethics Committee of Nanjing Ramda Pharmaceutical Co., Ltd. (No. IACUC-20220502).

### 2.4 Methods

#### 2.4.1 Synthesis of PDA NPs

DA (2 mg) was mixed in Tris-HCI (10 ml, 10 mM, pH 8.5) and stirred at room temperature using a magnetic stirring apparatus (500 rpm) for 8 h. Subsequently, the mixture was centrifuged at 12,000 rpm for 20 min using a centrifugal machine, washed several times with deionized water, then centrifuged at 12,000 rpm for 20 min, finally removed from the supernatant liquid, and the PDA NPs were collected.

#### 2.4.2 PDA-TMZ NPs synthesis

Because the metabolite of TMZ is Temozolomide acid *in vivo,* they have the identical cytotoxicity and antitumor effects. We selected Temozolomide acid to replace TMZ. Temozolomide acid (3 mg),NH_2_-PEG-NH_2_ Amine, (10 mg), and *N,N′*-dicyclohexylcar-bodiimide (2 mg) were mixed in anhydrous *N,N*-dimethylformamide (DMF, 1 mL).4-dimethylaminopyridine (2 mg) was then added, the mixture was reacted in an N_2_ atmosphere, and was stirred for 24 h at room temperature, and TMZ-PEG-NH_2_ was obtained. Next, TMZ-PEG-NH_2_ (3 mg) and PDA NPs (10 mg) were dissolved into the aqueous solution with vigorous stirring at room temperature. The mixture was then emulsified by ultrasonication for 10 min. Subsequently, the mixture was centrifuged at 14,000 rpm for 20 min and was washed thrice with water then continued to centrifuged at 14,000 rpm for 20 min to remove supernatant liquid and collect the PDA-TMZ NPs.

#### 2.4.3 Pep-1@PDA NPs synthesis

By adopting the above mentioned methods,Pep-1 (3 mg) was reacted with PDA NPs (10 mg) at 26°C to collect the Pep-1@PDA NPs.

#### 2.4.4 Pep-1@PDA-TMZ NPs synthesis

By adopting the above mentioned methods,Pep-1 (3 mg) and the PDA-TMZ NPs (10 mg) were mixed with an aqueous solution with vigorous stirring at 26°C to collect the Pep-1@PDA-TMZ NPs. Thereafter, FT-IR and UV-Vis were used for analyzing and characterizing.

#### 2.4.5 LR and EE of Pep-1@PDA-TMZ NPs

The loading rates (LR) and encapsulation efficiency (EE) of Pep-1@PDA-TMZ NPs were calculated by using UV-Vis. The different concentrations of TMZ (4,6,8,10, and 16 μg/mL) were prepared, and the standard curve of TMZ was drawn with DMF as the control to obtain the linear regression equation. The LR and EE were calculated using the following formula (Liu et al., 2013).

$$LR\left(\%\right)=\frac{W0-Wf}{W0}\times 100\%$$

$$EE\left(\%\right)=\frac{W0-Wf}{Ws}\times 100\%$$

where W_0_ represents the weight of the original TMZ,W_f_ represents the free TMZ, and Ws represents the weight of the complex after lyophilization.

#### 2.4.6 Biostability of NPs

To evaluate the stability of PDA NPs,PDA-TMZ NPs,Pep-1@PDA NPs, and Pep-1@PDA-TMZ NPs, we measured the potential of PDA NPs and Pep-1@PDA NPs by DLS. Meanwhile, water and 100% FBS were used to dissolve PDA NPs and Pep-1@PDA NPs. After 7 days, the transparency and clarification of PDA NPs and Pep-1@PDA NPs were verified respectively.

#### 2.4.7 pH-dependent release of TMZ

We added PDA-TMZ NPs and Pep-1@PDA-TMZ NPs in pH 5.0,4.0, and 7.4 PBS and then in succession shook at 25 °C respectively. The old PBS solution was replaced with a fresh one every 2 h, and the bulk volume of the solution remained unchanged. The supernatant liquid was tested by using UV-Vis. The typical absorption values of TMZ were utilized to draw the cumulative release graph of TMZ. The following formula was utilized to calculate the cumulative release rate (CRR) (Mei et al., 2020):

$$CRR\left(\%\right)=\frac{Total\;release\;mass\;of\;TMZ}{Total\;mass\;TMZ\;loaded\;in\;Pep-1@PDA-TMZ\;NPs\left(PDA-TMZ\;NPs\right)}\times 100\%$$

#### 2.4.8 Analysis of PTT conversion rate

To study the photothermal conversion efficiency of PDA NPs, PDA-TMZ NPs, Pep-1@PDA NPs, and Pep-1@PDA-TMZ NPs, the temperature variations of PDA NPs, PDA-TMZ NPs, Pep-1@PDA NPs, and Pep-1@PDA-TMZ NPs at different concentrations (set as 50, 100, 150, 200, and 250 μg/mL) were recorded under with 808 nm laser irritation. Furthermore, the temperature was recorded at different time points (set as 0, 1, 2, 3, 4, 5, 6, 7, 8, 9, and 10 min) *via* infrared thermography, for which water was set as the control group. The photothermal conversion efficiency (*η*) of the nanoparticles were calculated, according to reported method (Zhang et al., 2017).

#### 2.4.9 Endocytosis analysis of NPs

Human malignant glioma U87 cell lines were used to observe the cellular uptake of PDA-TMZ NPs and Pep-1@PDA-TMZ NPs using CLSM. The U87 cells were seeded into a 96-well plate at a density of 5 × 10^4^ cells per well, after 24 h incubation, U87 cells were incubated with PDA NPs, PDA-TMZ NPs, Pep-1@PDA NPs, and Pep-1@PDA-TMZ NPs at identical concentrations at 37°C for 1 h. To investigate the influences of incubation at different time points on U87 cellar uptake, U87 cells were incubated with the PDA-TMZ NPs and Pep-1@PDA-TMZ NPs (200 μg/mL) for 0.5, 1, 2, 4, 6, 8, 10, and 12 h at 37°C, respectively. The 96-well plates were stained with CCK-8 for 6 h. Finally, the cellular uptake dynamic behavior and outcome of PDA NPs, PDA-TMZ NPs, Pep-1@PDA NPs, and Pep-1@PDA-TMZ NPs were surveyed *via* CLSM.

#### 2.4.10 Cytotoxicity evaluation of NPs

U87 cells plated in a 96-well plates with a density of 1 × 10^4^ cells per well were incubated with the different concentrations (50, 100, 150, 200, and 250 μg/mL) of PDA NPs, PDA-TMZ NPs, Pep-1@PDA NPs, and Pep-1@PDA-TMZ NPs for 24 h. Subsequently, the experimental group was irradiated with the 808 nm laser (1 W/cm^2^) for 10 min, and the control group without irradiation. Then use CCK-8 to incubate for 6 h and then test cell viability. Optical density values were measured using a microplate reader to obtain cell viability data. For cytotoxicity experiments by CLSM U87 cells were seeded in confocal dishes and incubated for 24 h, and the control group without irradiation, followed by replacing the old culture with a medium containing different concentrations of PDA NPs, PDA-TMZ NPs, Pep-1@PDA NPs, and Pep-1@PDA-TMZ NPs (250 μg/mL each) for a further 6 h incubation. After 808 nm (1 W/cm^2^) laser irradiation for 10 min, staining was performed using 10 μL PI (1 mg/mL) and 10 µL of Calcein-AM (20 μg/mL) for 20 min. The CLSM images were obtained *via* Hoechst 33342.

#### 2.4.11 *In vivo* evaluation of live anticancer performance in mice

##### 2.4.11.1 Construction of the tumor nude mouse model

U87 cell were collected at 5 × 10^7^/mL density. By subcutaneous inoculation, 0.1 ml was implanted in the right axilla of each mouse, and the experiments were performed when the tumor volume of all mice were grown for 3 weeks.

##### 2.4.11.2 Anesthesia time and method

When the tumor volume of all mice grew to approximately 80 mm^3^, the anesthesia method was inhalation, and the narcotic drug was 2.5% isoflurane.

##### 2.4.11.3 Therapeutic schedule

When the tumor volume in each of the 40 mice grew to approximately 80 mm^3^, the tumor-bearing nude mice were freely divided into eight groups, and each had five tumor-bearing nude mice, then respectively injected *via* the caudal vein with 150 μl of saline, Pep-1@PDA NPs, PDA-TMZ NPs, and Pep-1@PDA-TMZ NPs 12 h later, the tumor site of corresponding groups were exposed to 808 nm laser for 10 min (1 W/cm^2^). The concentration of coumarin-6, TMZ, PDA, and Pep-1 were 200 200, 200, and 100 μg/mL, respectively.When all trials were completed, the nude mice were killed by euthanasia with carbon dioxide. The tumor slices of mice treated with saline, Pep-1@PDA NPs, PDA-TMZ NPs, and Pep-1@PDA-TMZ NPs at 24 h after irradiation were stained with hematoxylin and eosin (H&E), moreover, the H&E images of major organs were obtained after 14 days of treatment.

##### 2.4.11.4 Observational indicators

To collect the experimental data, the day of drug injection was recorded as day 0. The body weight and tumor volume (TV) of each mouse were measured every second day. TV was calculated using the following equation.

$$TV=½\times a\times b^{2}$$

where **a** and **b** represent the long and short diameters (in mm),respectively.

The relative tumor volume (RTV) were calculated using the following formula:

$$RTV=V_{t}/V_{0}$$

where V_0_ is the TV, as calculated by separate-cage administration, and V_t_ is the TV, as calculated at each measurement.

The tumor growth inhibition rate was calculated as follows:

$$Inhibition\;rate\left(\%\right)=\frac{Mean\;tumor\;weight\;of\;the\;control\;group-Mean\;tumor\;weight\;of\;the\;treatment\;group}{Meant\;tumor\;weight\;of\;the\;control\;group}\times 100\%$$

Pathological sections of tumor tissue, H&E staining, and routine blood examination were utilized to verify the safety of NPs.

##### 2.4.11.5 Statistical analysis

All experimental results were expressed as mean ± standard deviation and one-way ANOVA was conducted using GraphPad Prism software (version 6.0; GraphPad, San Diego, CA, United States), and statistical significance was considered at a ***p***-value <0.05.

## 3 Results

### 3.1 Morphological characterization of NPs

**FIGURE 3:**
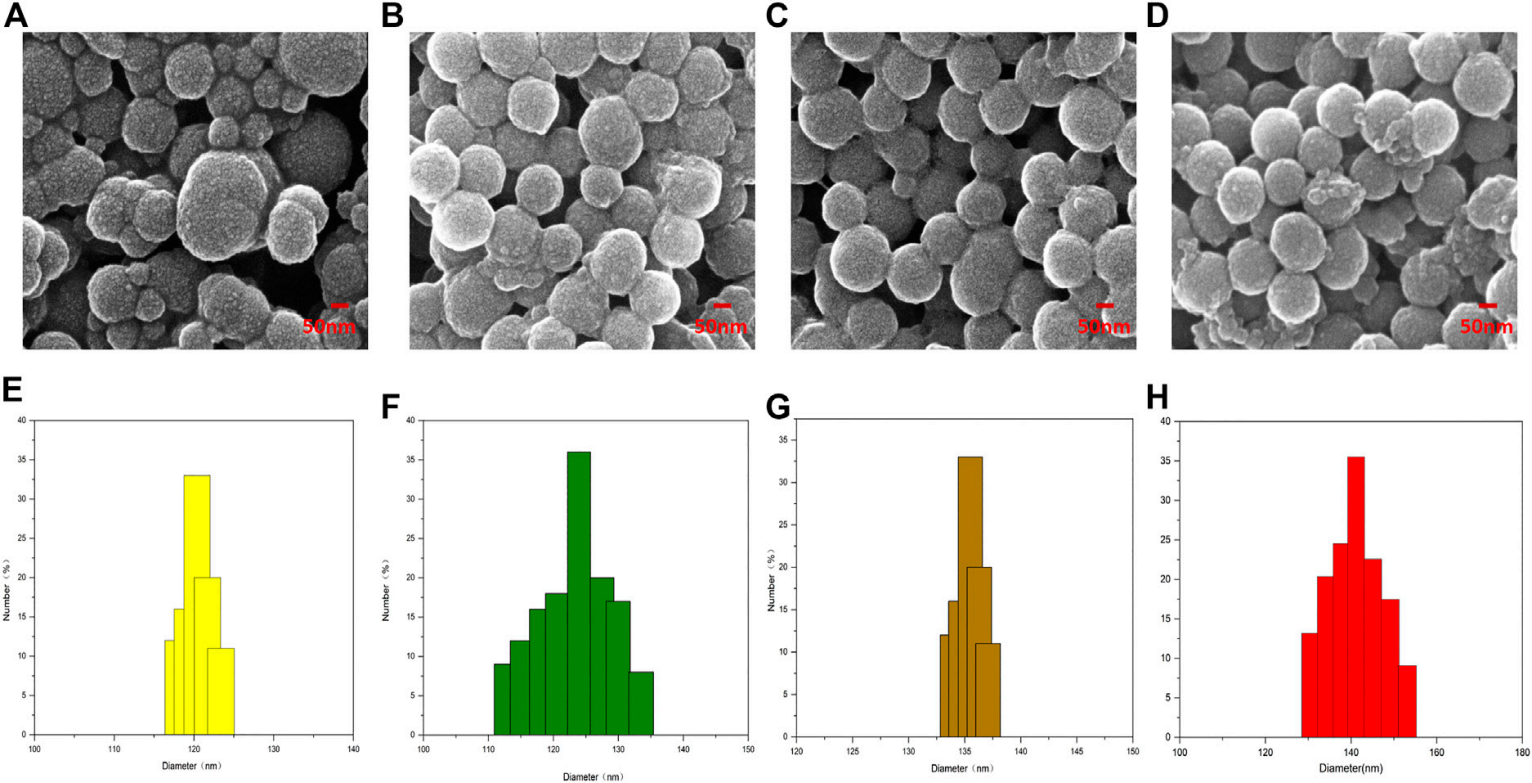
The Characterization of different NPs: **(A–D)** SEM **(E–H)**; The size and distribution of different NPs.

The shape of PDA NPs, PDA-TMZ NPs, Pep-1@PDA NPs, and Pep-1@PDA-TMZ NPs were regular and spherical, and the characterization analysis of DLS and SEM indicated that the dimensions of the above NPs were approximately 120, 125, 135, and 140 nm, respectively, with well-proportioned dimensions and smooth surfaces that could be lightly traverse and could attach to the tumor location *via* the enhanced permeability and retention (EPR) effect (Zheng et al., 2020). The Pep-1@PDA-TMZ NPs dimensions gradually increased, indicating that TMZ and Pep-1 were successfully loaded (Figure 3).

### 3.2 Assembly analysis of Pep-1@PDA-TMZ NPs

The chemical composition of the Pep-1@PDA-TMZ NPs was analyzed by UV-Vis and FT-IR. The FT-IR of Pep-1@PDA-TMZ NPs possessed new peak value at 1626, 3421, and 2,912 cm^−1^, which conformed to Pep-1 and TMZ. Above relatively typical peaks value indicated that Pep-1 and TMZ had been successfully conjugated to the PDA NPs (Supplementary Figure S1A). Moreover, the peak value of TMZ (1,699 cm^−1^) and that of Pep-1 (1,683 cm^−1^) merged into a single peak at 1,626 cm^−1^, signifying the formation of the Sbb (C = N). Furthermore, the UV-Vis spectrum verified the typical peak value of TMZ at 329 nm, Pep-1 at 229 nm, and PDA at 285 nm. Meanwhile, Pep-1@PDA-TMZ NPs demonstrated relatively broader absorption at 329 nm (Supplementary Figure S1B). Therefore, the assembly of the carbonyl group of Pep-1and TMZ with the PDA amino group formed Sbb (C=N). Therefore, we speculated that the SBR have formed.

### 3.3 Stability of the Pep-1@PDA NPs

**FIGURE 4:**
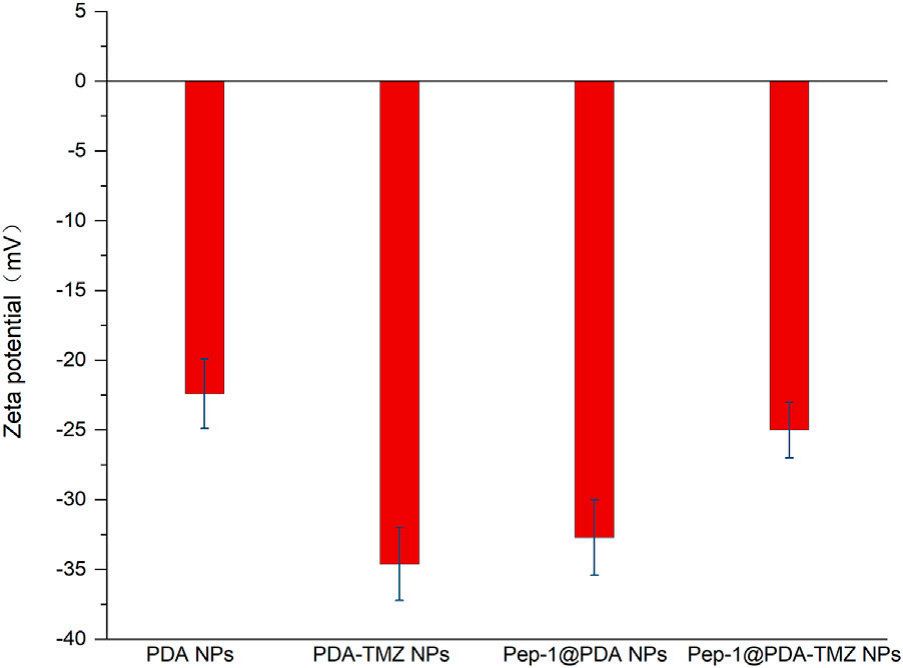
The zeta potential of the different NPs.

The stability of NPs are of significance for antitumor treatment. The zeta potential of PDA NPs,PDA-TMZ NPs, Pep-1@PDA NPs and Pep-1@PDA-TMZ NPs was approximately −22.4, −34.6, −32.7, and −25 mV, respectively (Figure 4), which can ensure these PDA NPs, PDA-TMZ NPs, Pep-1@PDA NPs, and Pep-1@PDA-TMZ NPs should not be eliminated easily in the body circulation. Afterwards, PDA NPs and Pep-1@PDA NPs were dissolved in water and FBS to carefully observe their dissolution. After 7 days, the solutions maintained a homogeneous brightness. Thereby, the results proved that the solubility and stability of PDA NPs and Pep-1@PDA NPs were relatively good (Supplementary Figure S2).

### 3.4 Encapsulation and release of TMZ

To calculate the LR and EE of PDA-TMZ NPs and Pep-1@PDA-TMZ NPs, TMZ (10 μg/mL) was dissolved to DMF and analyzed by UV-Vis spectroscopy, which denoted that the standard absorption peak value of TMZ occurred at approximately 329 nm (Supplementary Figure S3A), and this peak value can be utilized to calculate the content of TMZ. Supplementary Figure S3B presented the absorbance of different concentrations of TMZ. The EE and LR of PDA-TMZ NPs and Pep-1@PDA-TMZ NPs were approximately 48.5% and 10.3%, 50%, and 10.40%, respectively.

**FIGURE 5:**
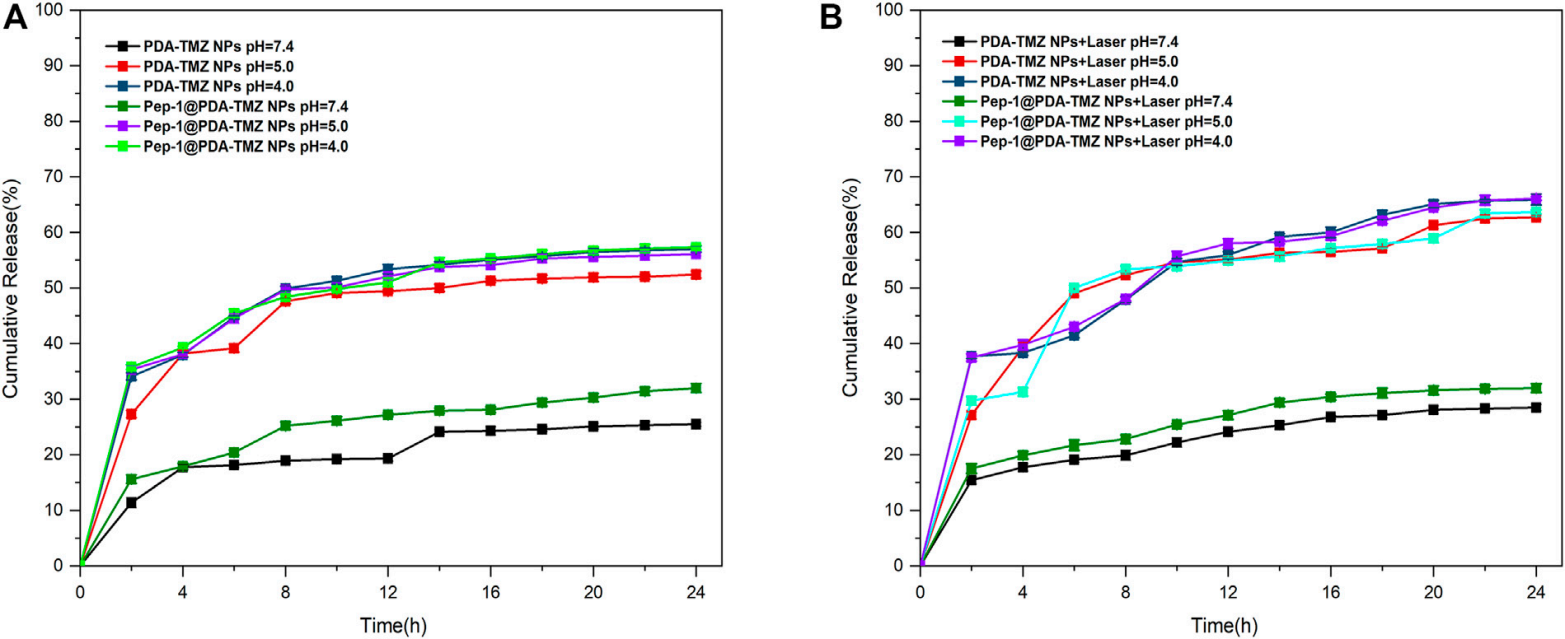
**(A)** TMZ release curve of the PDA-TMZ NPs in different pH values of PBS. **(B)** TMZ release curve of the Pep-1@PDA-TMZ NPs with 808 nm laser irradiation in different pH values of PBS.

The trials verified that the Sbb C = N of PDA-TMZ NPs and Pep-1@PDA-TMZ NPs can be break, to cause the release of TMZ in certain acidic environments. To prove pH-dependent respond release of PDA-TMZ NPs and Pep-1@PDA-TMZ NPs loaded with TMZ, pH = 7.4, 5.0, and 4.0 were utilized to simulate the normal physiological condition and acidic TME. When pH = 7.4, the total cumulative release rate (CRR) of TMZ of PDA-TMZ NPs and Pep-1@PDA-TMZ NPs over 24 h was approximately 25.5% and 32%, and when pH = 5.0, CRR was approximately 52.4% and 56.1%. However, interestingly, when pH = 4.0, CRR reached approximately 57% and 57.3% (Figure 5A). Therefore, PDA-TMZ NPs and Pep-1@PDA-TMZ NPs can cause more Sbb C=N breakage under the acidic TME. PDA-TMZ NPs and Pep-1@PDA-TMZ NPs have lower adverse effects on normal cells and are safe, trustworthy, and efficient in antitumor activity.

To investigate whether PTT therapy in the acidic environment has a good impact on TMZ release from PDA-TMZ NPs and Pep-1@PDA-TMZ NPs.The results proved that the release rate of TMZ can be enhanced considerably to about 65.9% and 66.1% *via* 808 nm laser irradiation (Figure 5B). Hence, the, PTT effect of PDA-TMZ NPs and Pep-1@PDA-TMZ NPs could cause the dissociation of more Sbb C=N and accelerate the release of TMZ The PDA-TMZ NPs and Pep-1@PDA-TMZ NPs can serve as nano-drug delivery systems against GBM and should be good pH-responsive drug-release nanocarriers.

### 3.5 PTT conversion rate of NPs

**FIGURE 6:**
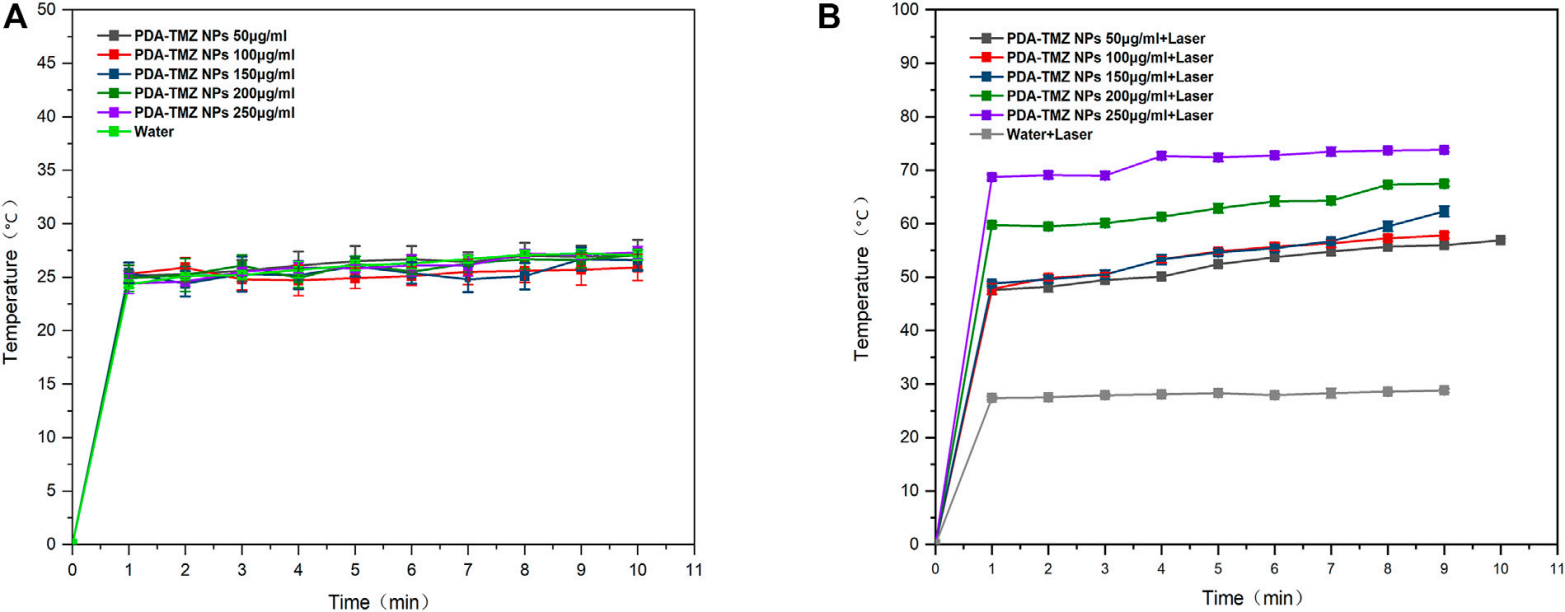
**(A)** The temperature variation of the PDA-TMZ NPs (different concentrations). **(B)** The temperature variation of PDA-TMZ NPs (different concentrations with laser).

**FIGURE 7:**
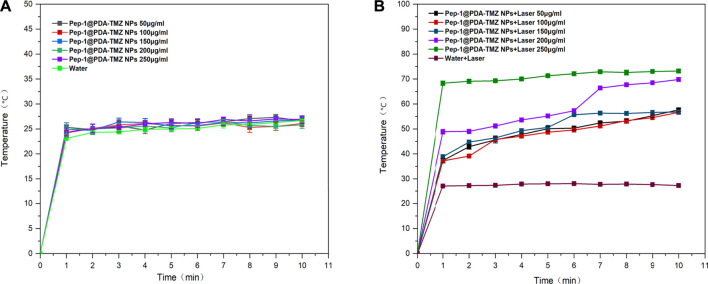
**(A)** The temperature of the Pep-1@PDA-TMZ NPs (different concentrations). **(B)** The temperature of the Pep-1@PDA-TMZ NPs (different concentrations with laser).

After 10 min of 808 nm laser irradiation, the PTT was assessed, and the temperature variation at different the PDA-TMZ NPs and Pep-1@PDA-TMZ NPs concentrations were tested using thermography. The temperature of the PDA-TMZ NPs and Pep-1@PDA-TMZ NPs solution rose from 33.7°C to 73.8°C, and 33°C–72.6°C (Figure 6). But, regrettably, the temperature of the PDA-TMZ NPs and Pep-1@PDA-TMZ NPs almost did not change without laser irradiation, and almost close to room temperature (Figure 7). Therefore, the PDA-TMZ NPs and Pep-1@PDA-TMZ NPs exhibited relatively better PTT effects. The photothermal conversion efficiency of the Pep-1@PDA NPs was further calculated to be 49.2%, according to previous reported method (Supplementary Figure S4) (Zhang et al., 2017).

### 3.6 Endocytosis analysis

**FIGURE 8:**
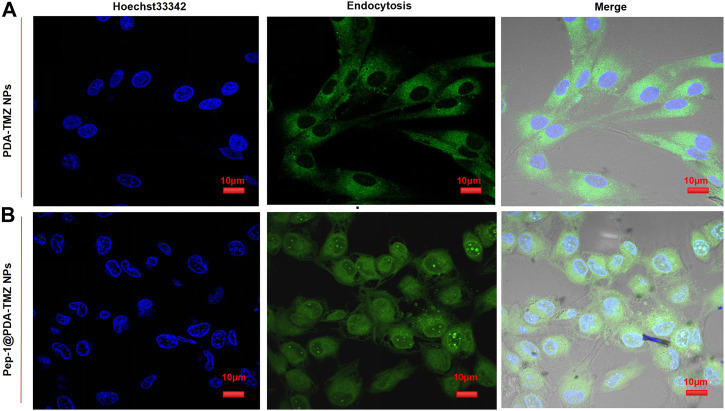
**(A)** The PDA-TMZ NPs gathered in the cytoplasm of U87 cells. **(B)** The Pep-1@PDA-TMZ NPs were endocytosed by the nucleus of U87 cells (green fluorescence).

The endocytosis of PDA-TMZ NPs and Pep-1@PDA-TMZ NPs was explored. PDA-TMZ NPs and Pep-1@PDA-TMZ NPs in U87 cells were evaluated by using CLSM (Figure 8). The cells were stained and showed blue fluorescence. The drug-loaded NPs displayed green fluorescence after entering U87 cells. After co-incubation for 6 h with U87 cells, Pep-1@PDA-TMZ NPs were successfully endocytosed into the nucleus of U87 cells. However, the PDA-TMZ NPs failed to endocytose into nucleus of U87 cells, but they only stayed around the cytoplasm. Thus, the PDA-based NPs modified with Pep-1 had a strong affinity and penetrating power in U87 cells and could enhance the effectiveness of antitumor therapy by carrying chemotherapy drugs. Therefore, the Pep-1@PDA-TMZ NPs have wide potential for tumor-targeted therapy.

### 3.7 Cytotoxicity evaluation

**FIGURE 9:**
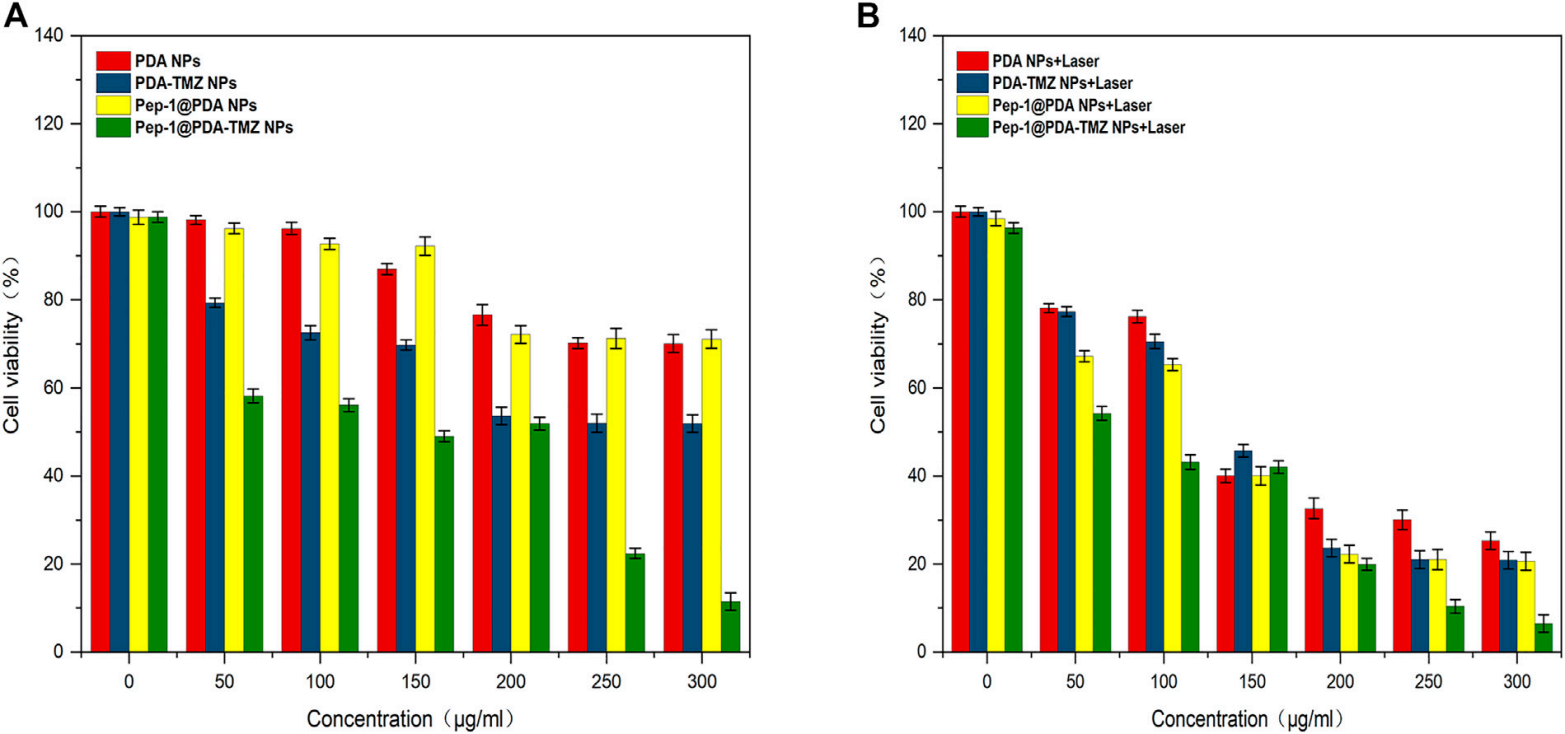
**(A)** The inhibition rate of the different NPs (without Laser). **(B)** The inhibition rate of the different NPs (with laser).

After incubation for 6 h, CCK-8 was used for analyzying NPs activity in cells. The associated cytotoxicity of PDA NPs, PDA-TMZ NPs, Pep-1@PDA NPs, and Pep-1@PDA-TMZ NPs to U87 cells were tested. As revealed in Supplementary Figure S5, the PDA NPs and Pep-1@PDA NPs had slight impact on the activity of U87 cells. Above results further indicated that the particles possessed good biocompatibility and had no obvious toxicities or adverse effects. U87 cells with at different concentrations of PDA-TMZ NPs and Pep-1@PDA-TMZ NPs were incubated for 6 h. The experimental group was irradiated with an 808 nm laser for 10 min, while the control group was not irradiated. Post treatment, the result proved that the inhibition rate of the experimental groups were more prominent than the control groups, hence proving the associated antitumor effect of CT and PTT. Furthermore, cytotoxicity to cancer cells was obviously augmented with increasing the dosage of TMZ, which demonstrated dosage positive correlation. When the dosage of the PDA-TMZ NPs and Pep-1@PDA-TMZ NPs were increased to 300 μg/mL and were associated with PTT therapy, the inhibition rates in U87 cells were approximately 80% and 94%, respectively (Figures 9A, B). The therapeutic effects of the Pep-1@PDA-TMZ NPs were relatively better than those of the PDA-TMZ NPs after co-incubation with U87 cells for 6 h. Thus, the PDA-TMZ NPs conjugated by Pep-1 were more prominent against malignant GBM cells and could further promote the effect and activity of antitumor.

**FIGURE 10:**
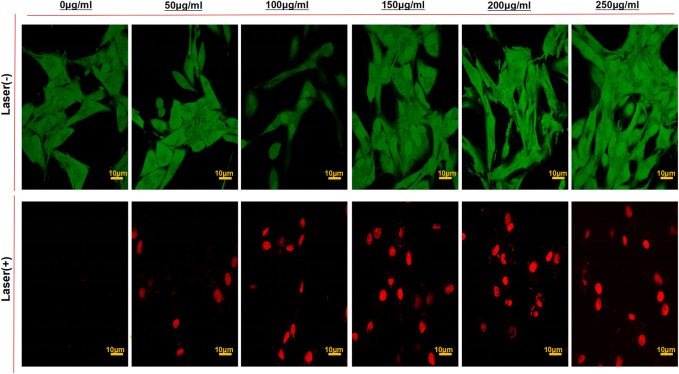
The CLSM images of U87 cells stained with Calcein-AM/PI to visualize cell viabilities treated by the PDA-TMZ NPs and Pep-1@PDA-TMZ NPs with or without 808 nm laser (Green:live; Red:death).

To further inspect the antitumor activity of Pep-1@PDA-TMZ NPs *in vitro*, the viability of U87 cells (staining of live dead cells) was assessed using CLSM (Figure 10). After U87 cells incubated with the different dosage of the Pep-1@PDA-TMZ NPs combined with 808 nm laser irradiation indicated the survival rates of U87 cells markedly reduced than without irradiation. Thus, the Pep-1@PDA-TMZ NPs presented stronger CT and PTT, which offer more confidences and evidences to us in the subsequent *in vivo* trials.

### 3.8 *In vivo* anticancer effect of Pep-1@PDA-TMZ NPs

**FIGURE 11:**
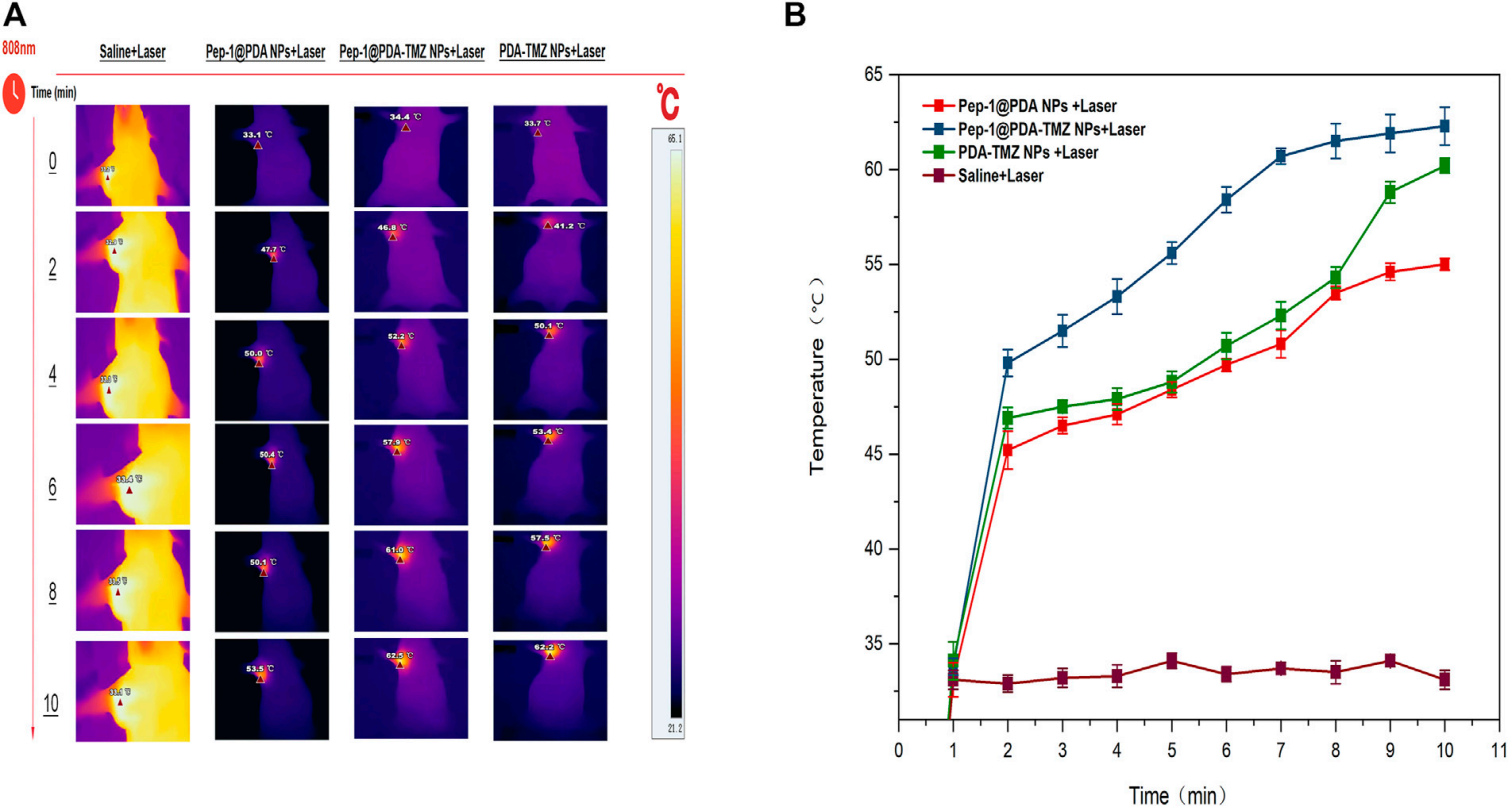
**(A)** The thermal images and **(B)** temperature rise curves of tumor sites in tumor-bearing nude mice within 10 min post laser irradiation.

**FIGURE 12:**
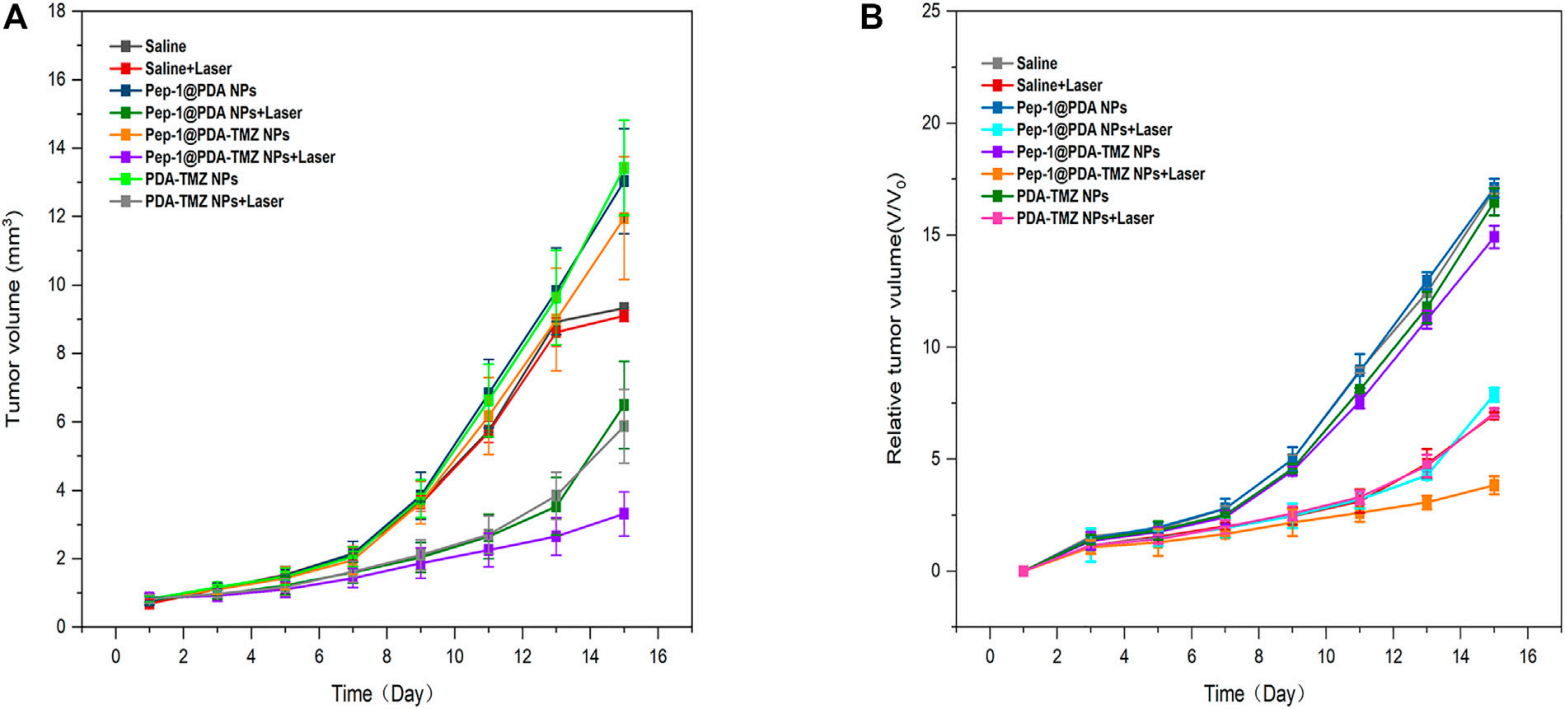
**(A)** The tumor volume change of tumor-bearing mice after different treatments denoted. **(B)** Relative tumor volume of tumor-bearing mice after different treatments denoted.

**FIGURE 13:**
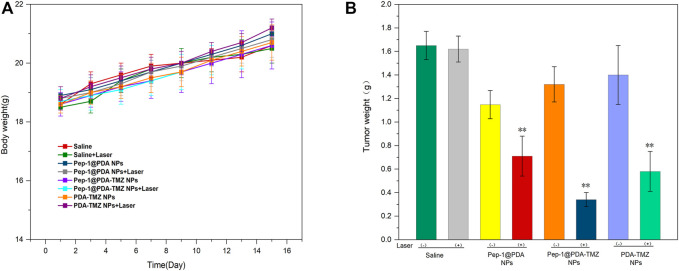
**(A)** The body weight of the tumor-bearing mice after different treatments. **(B)** Tumor weight of the tumor-bearing mice after different treatments.

In consideration of the antitumor therapeutic effect of Pep-1@PDA-TMZ NPs was relatively satisfying *in vitro*, the antitumor effect of an integration therapy of CT and PTT of the Pep-1@PDA-TMZ NPs was verified utilizing U87 cells in tumor-bearing nude mice *in vivo*. When the mice TV increased to approximately 80 mm^3^, Pep-1@PDA-TMZ NPs (200 μg/mL, 150 μL) were injected into the nude mice *via* the caudal vein. The PDA-TMZ NPs and Pep-1@PDA NPs were set as the control groups. The temperatures at the tumor site of nude mice injected with the Pep-1@PDA-TMZ NPs were promptly increased to approximately 60°C within 7 min (Figure 11). The antitumor therapeutic effect of nude mice denoted that the growth of tumors treated with Pep-1@PDA NPs was slightly inhibited without laser irradiation. In comparison, the growth of tumors treated with PDA-TMZ NPs and Pep-1@PDA-TMZ NPs with laser irradiation was considerably inhibited due to the association of CT and PTT, but Pep-1@PDA-TMZ NPs was relatively superior. In addition, the safety of Pep-1@PDA-TMZ NPs was evaluated *via* with an *in vivo* antitumor activity analysis. Finally, the volume of tumors and RTV of nude mice in each group was monitored (Figures 12A, B). During the experiment, the body and tumor weight of mice in all groups increased (Figures 13A, B), thereby validating the good biocompatibility of the Pep-1@PDA-TMZ NPs.

**FIGURE 14:**
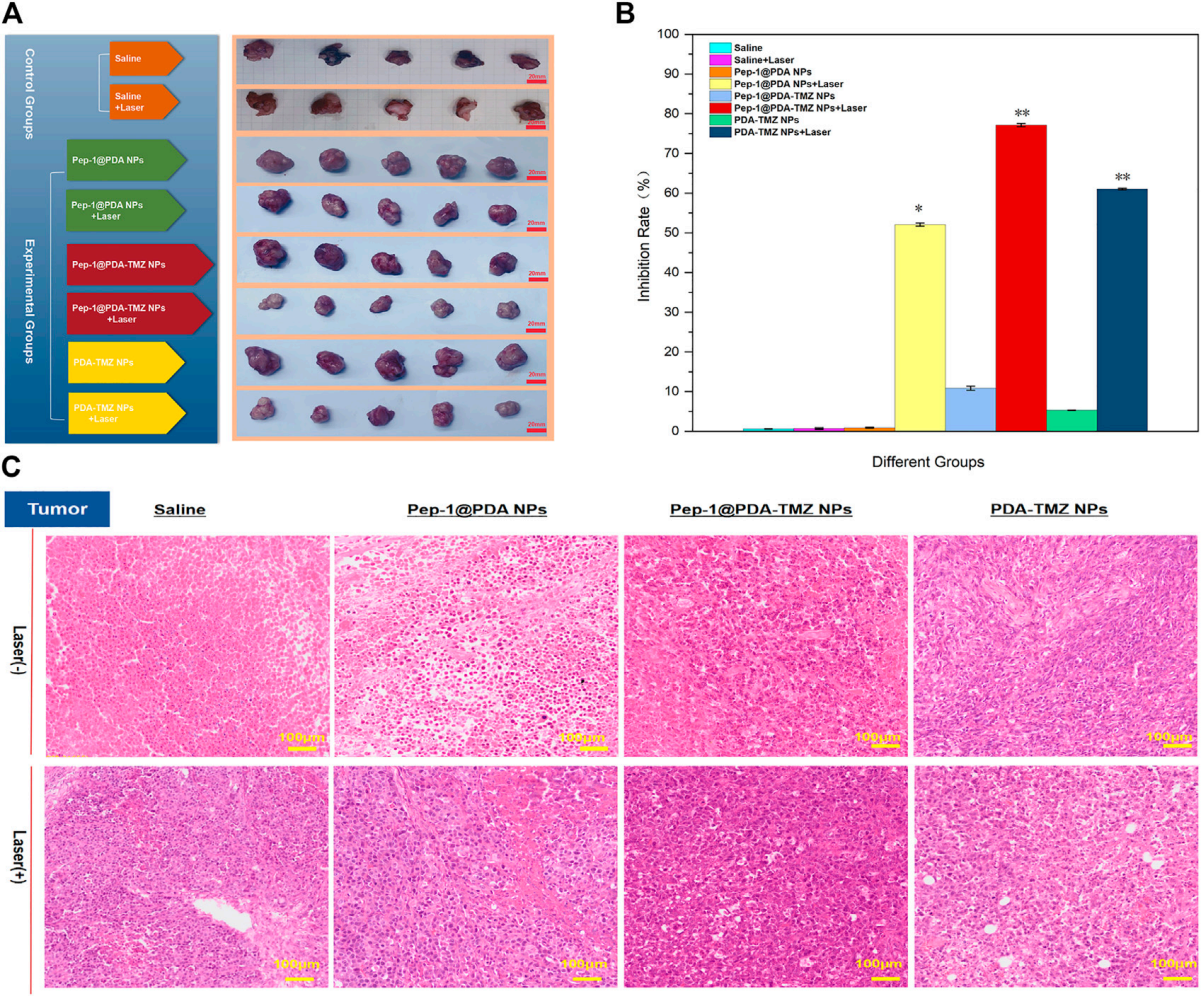
**(A)** *In vivo* PTT combined with CT efficacy of the NPs. **(B)** The tumor inhibition by different groups of drugs. *p < 0.05,**p < 0.01; **(C)** The H&E images of tumor treated with the Saline, Pep-1@PDA NPs, Pep-1@PDA-TMZ NPs, and PDA-TMZ NPs at 24 h post irradiation (808 nm, 1 W/cm^2^, and 10 min).

In conclusion, compared with the other groups,Pep-1@PDA-TMZ NPs with Laser had the best antitumor performance (Figures 14A, B).Figure 14C presented the H&E staining of tumor site. The images depicted that no obvious apoptosis occurred in the Pep-1@PDA NPs and Pep-1@PDA NPs + Laser groups or in the PDA-TMZ NPs groups, whereas PDA-TMZ NPs + Laser and Pep-1@PDA-TMZ NPs groups indicated partial apoptosis. The Pep-1@PDA-TMZ NPs + Laser denoted evident apoptosis and rupture.

**FIGURE 15:**
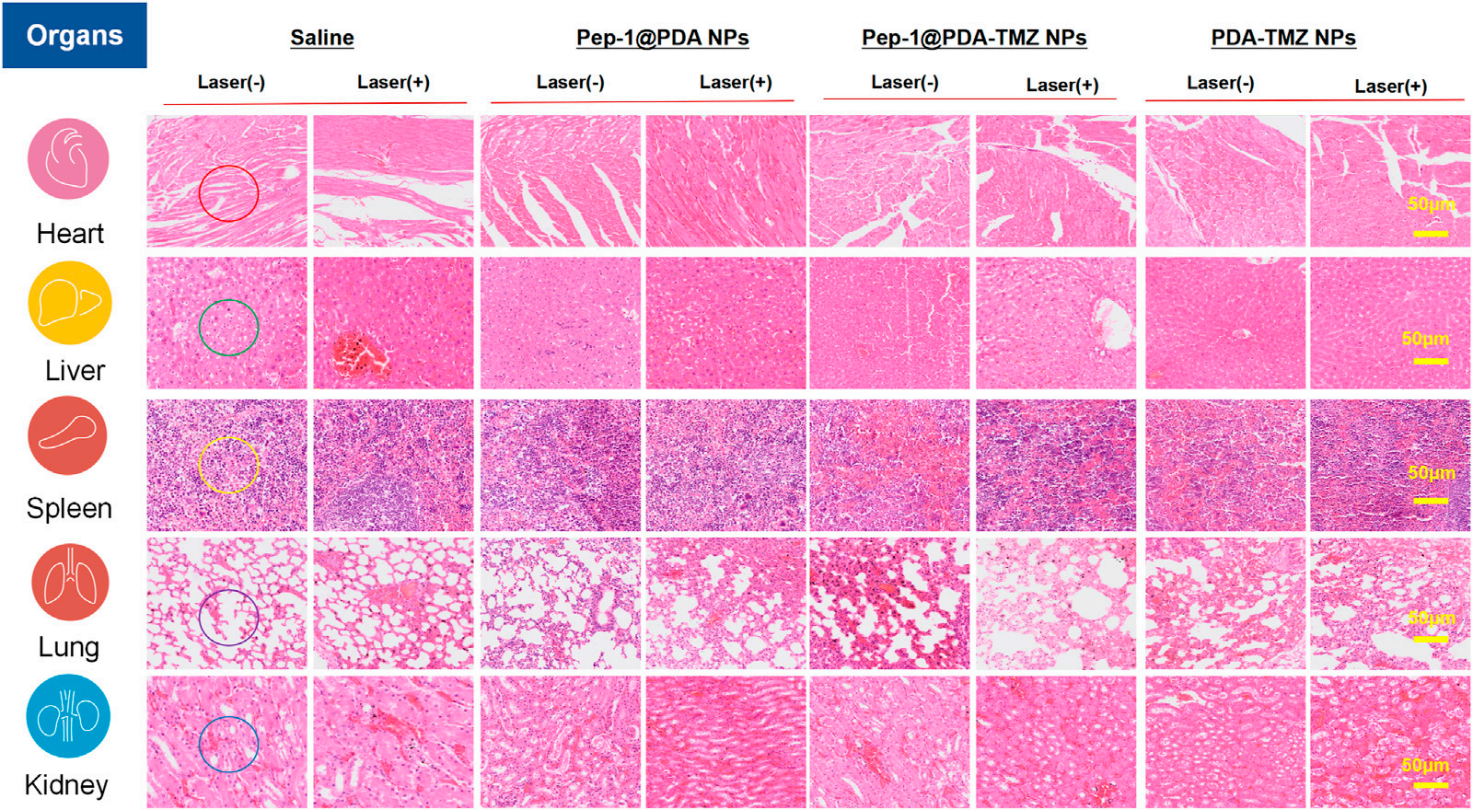
H&E staining of the major organs in nude mice. After treatment of Saline, Pep-1@PDA NPs, Pep-1@PDA-TMZ NPs, and PDA-TMZ NPs Heart: The myocardial fibers are aligned (red circle); liver: No significant vacuolization (green circle), spleen: No obvious irregular shape of white pulp (yellow circle); lung: No obvious lymphocyte infifiltration (purple circle) and kidney: No obvious nephrotoxicity (bule circle).

To systematically investigate the toxicity of Pep-1@PDA-TMZ NPs toward major organs in nude mice, the H&E stained assay of each organ was conducted. There was no visible damage to the heart (no cardiac muscle fibers disorder), liver (no significant vacuolization), spleen (no obvious irregular shape of white pulp), lung (no obvious lymphocyte infifiltration), or kidney tissues (no obvious nephrotoxicity) in nude mice of Pep-1@PDA-TMZ NPs (Figure 15) and no significantly alter white blood cells, lymphocytes, neutrophils, platelet count, or hemoglobin level. Supplementary Figure S6 displayed blood routine examination of post-treatment nude mice. These results indicated the safety and biocompatibility of Pep-1@PDA-TMZ NPs for GBM treatment. Nevertheless, further investigation should be conducted to ensure the *in vivo* safety and biocompatibility of Pep-1@PDA-TMZ NPs.

## 4 Discussion

In clinical practice, the traits of GBM include high mortality and low postoperative survival. The standard of the treatment is surgery combined with RT and CT. But the overall therapeutic effects are extremely frustrating, meanwhile, side-effects associated with therapies were severe (Thaci et al., 2014).

Photothermal therapy (PTT) is an emerging physicochemical treatment method with high selectivity and minimal invasiveness, in which light energy is converted to thermal energy *via* near-infrared light irradiation from an external auxiliary light source. The rapidly increasing temperature is used to conquer targeted tumor cells through photothermal effects. PDA can be used as a photothermal therapeutic agent, the research shown that PDA had no significant damage to the surrounding normal tissues and with reduced side effects. This study demonstrated the superior photothermal effects of PDA. We found the prepared stable and well-dispersed PDA-based NPs with an advanced photothermal conversion rate of 49.2%. The dispersion temperature of these PDA-based NPs was rapidly increased by 73.8°C *via* 808 nm laser irradiation (1 W/cm^2^) for 10 min at a concentration of 200 μg/mL in U87 cells. PDA-based NPs are an appropriate drug delivery system for high-efficiency loading of antitumor drugs with improved side effect (Milletti, 2012; Guidotti et al., 2017; Farokhi et al., 2019; Dai et al., 2021). PDA contains amino groups and catechol that can encapsulate other materials, including TMZ, Doxorubicin (DOX), and paclitaxel (PTX) (Guidotti et al., 2017). Additionally, its advantages include excellent adhesion capabilities, PTT effects, PDT effects, good the photothermal conversion efficiency (compare with some reported PDA nanoparticles, such as PSBTBT@PDA NPs,PDA-PEG/DOX (Wang et al., 2016))) and pH-responsive drug-release properties (Liu et al., 2014; Shao et al., 2020). The photothermal therapy of PDA can be combined with photodynamic therapy to treat tumors. In photodynamic therapy, photosensitizers can convert molecular oxygen (O_2_) into cytotoxic reactive oxygen species (ROS), especially singlet oxygen (^1^O_2_), which can kill tumor cells. However, up to now, only a few studies have reported the combination of photothermal therapy of polydopamine-based and photodynamic therapy for tumor treatment. Hence, PDA can become a potential nanocarrier for antitumor therapy (Liu et al., 2014), but the PDA-based NPs are inefficient for active tumor targeting.

The current study has confirmed that almost all GBM highly express the IL-13 receptor. IL-13Rα2, a monomer of IL-13, is a highly affinity receptor encoded by the X chromosome and consists of 380 amino acids, 17 amino acid domains and 26 amino acid signal sequences. However, IL-13Rα2 was rarely expressed in normal brain cells but highly expressed in GBM. This specificity makes it an ideal target for therapeutic in GBM. Some studies also confirmed that Pep-1 is a peptide ligand capable of highly specific binding to IL-13Rα2 and has the highest affinity. Furthermore, the expression of the IL-13Rα2 receptor has also been shown to harbor in tumor-infiltrating cells, tumor-initiating cells, or GSCs and neovascularization (Knudson et al., 2022). Research of Wang indicated that Pep-1 can invade glioma tumor cell nuclei *via* IL-13Rα2-mediated endocytosis (Wang et al., 2019). Lin also demonstrated that IL-13Rα2 was over-expressed in GBM and that a nano-delivery system constructed utilizing Pep-1 was efficient at impeding tumor proliferation *via* the regulation of the JNK signaling pathway (Lin et al., 2021). In our work,Pep-1carried NPs could permeate U87 cells and enter the cell nucleus *via* transmembrane mechanisms (Pandya et al., 2012; Wang et al., 2015; Jiang et al., 2016; Lv et al., 2016; Jiang et al., 2017; Jiao et al., 2017; Wang et al., 2017; Guo et al., 2019; Wang et al., 2020; Choi et al., 2021) According to cell experiments, we tracked the endocytosis of PDA-TMZ NPs and Pep-1@PDA-TMZ NPs by coumarin-6 labeling. The results showed that Pep-1@PDA-TMZ NPs were successfully endocytosed into the nucleus of U87 cells. However, the PDA-TMZ NPs failed to endocytose into nucleus of U87 cells, but they only stayed around the cytoplasm. We speculate that the Pep-1@PDA-TMZ NPs could be targeted to GBM *via* IL-13Rα2-mediated endocytosis, However, the specific transmembrane mechanisms of Pep-1 carried NPs need to be further explored in the future research, including the involvement of other signaling pathways.

In conclusion, Pep-1 was conjugated and modified to the PDA@TMZ NPs, which can not only afford an integration treatment of CT and PTT therapy against GBM, but can also enhance the velocity and accuracy of targeted entry into tumor location, indicating that the Pep-1@PDA-TMZ NPs combined with PTT therapy are more effective than single CT or PTT treatment at gradually wiping out tumor tissues, suggesting that Pep-1@PDA-TMZ NPs could be a promising approach as novel, targeted,nano-delivery systems for GBM therapy and further offer some evidence and data for clinical transformation and so that gradually conquer GBM.

## Data Availability

The raw data supporting the conclusion of this article will be made available by the authors, without undue reservation.

## Abbreviations

CBTRUS, central Brain Tumour Registry of the United States; CT, chemotherapy; DA, dopamine; DCC, N,N’dicyclohexylcarbodiimide; DLS, dynamic Light Scattering; DMAP, 4-dimethylaminopyridine; DMF, N,N-dimethylformamide; EE, encapsulation efficiency; EPR, enhanced permeability and retention; FBS, fetal bovine serum; GBM, glioblastoma multiforme; GSCs, glioma stem cells; H&E, hematoxylin andeosin; IL-13Rα2, interleukin-13 receptor α2; LR, loading rate; MGMT, O6-methylguanine-DNA methyltransferase; NIR, near-infrared; NPs, nanoparticles; PDA, polydopamine; PEG, polyethylene glycol; Pep-1, peptide-1; PTT, photothermal; SEM, scanning Electron Microscope; TEM, transmission Electron Microscope; TMZ, temozolomide; UV-Vis, ultraviolet-visible.
