# Supplementary figures and images for: Polydopamine-based loaded temozolomide nanoparticles conjugated by peptide-1 for glioblastoma chemotherapy and photothermal therapy
Source: Front Pharmacol. 2023 Jan 18;14:1081612. doi: 10.3389/fphar.2023.1081612 (PMC9889548; doi:10.3389/fphar.2023.1081612)

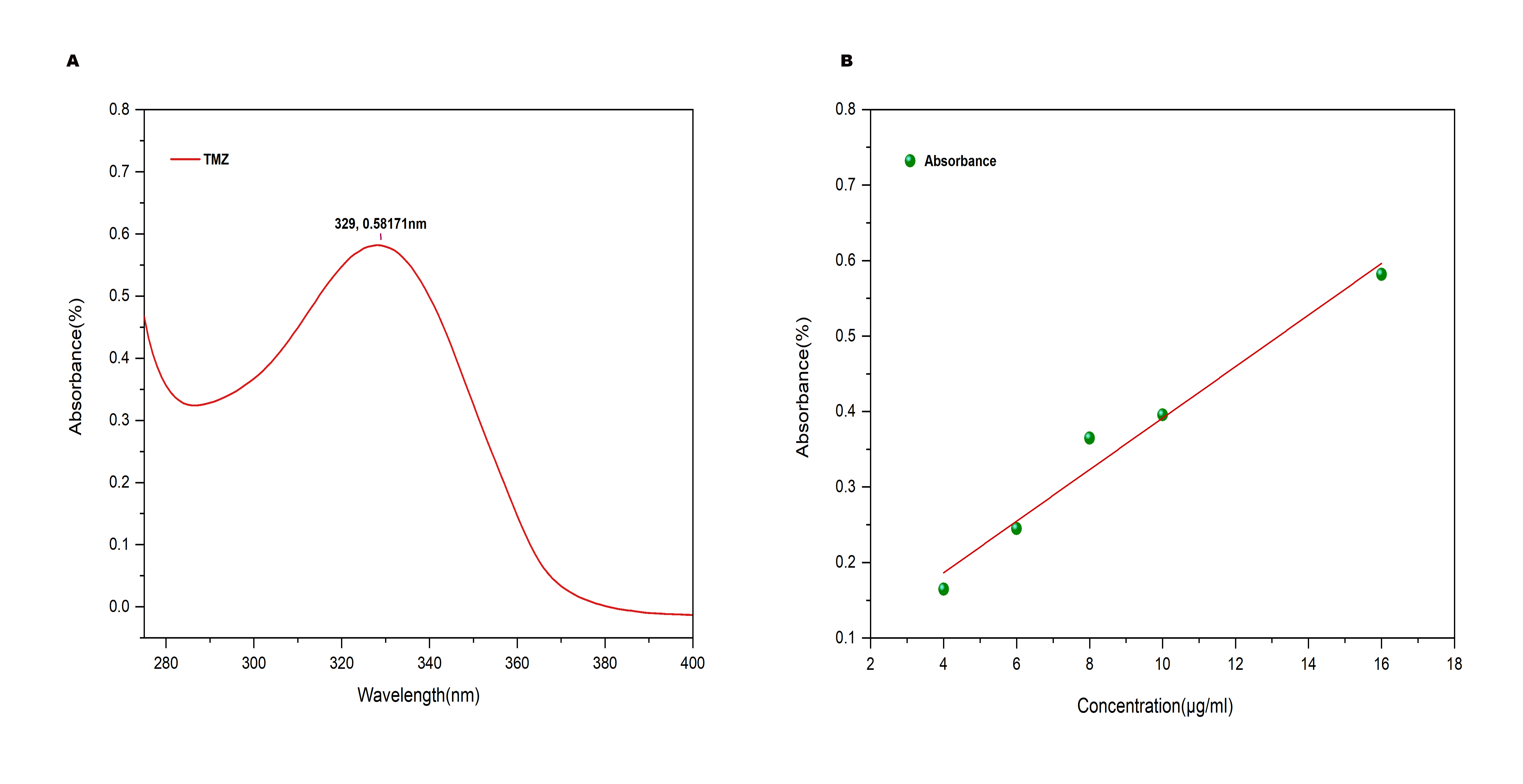

Supplement: Supplementary file 2 [file Image3.TIF]

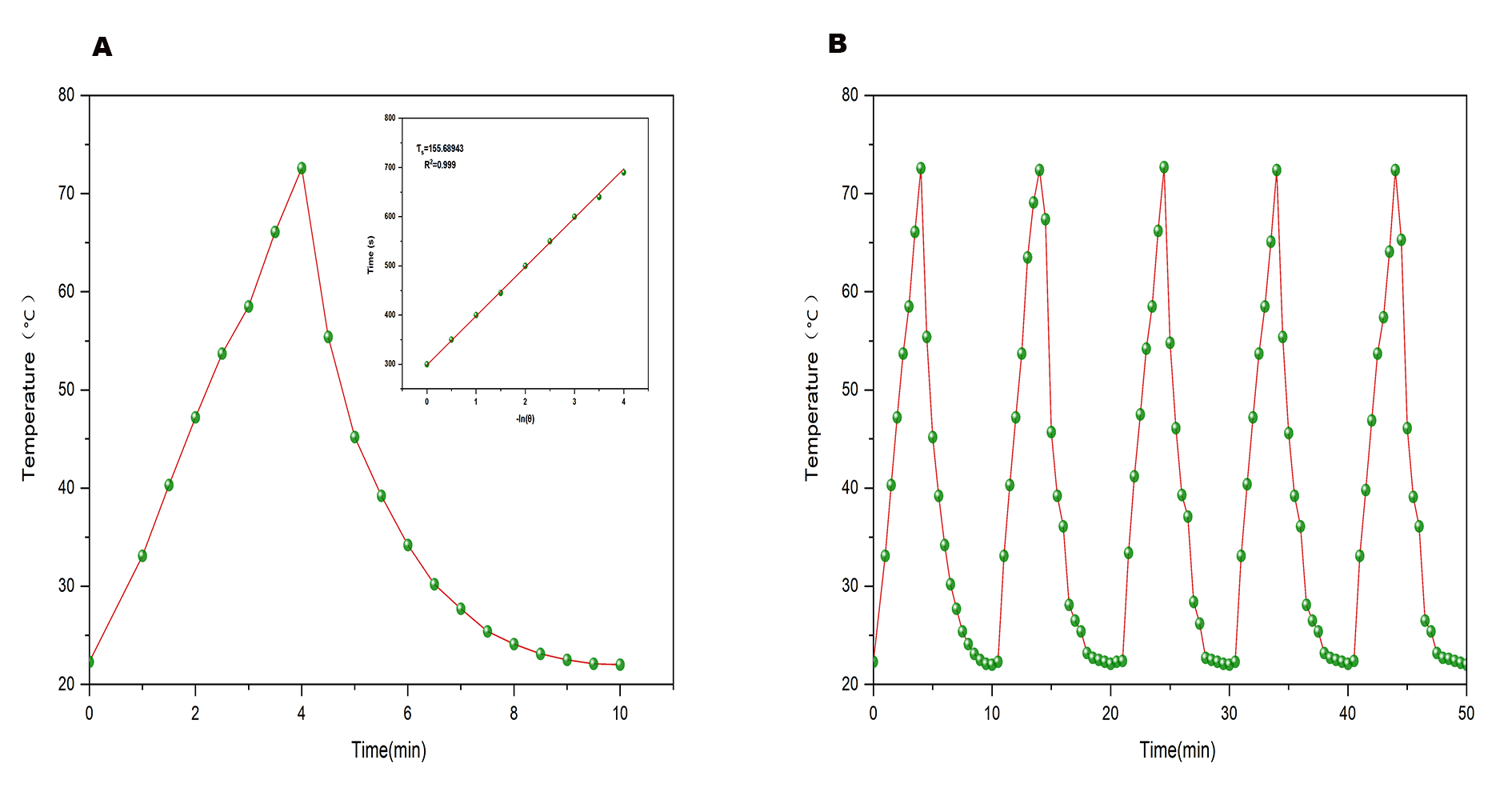

Supplement: Supplementary file 3 [file Image4.TIF]

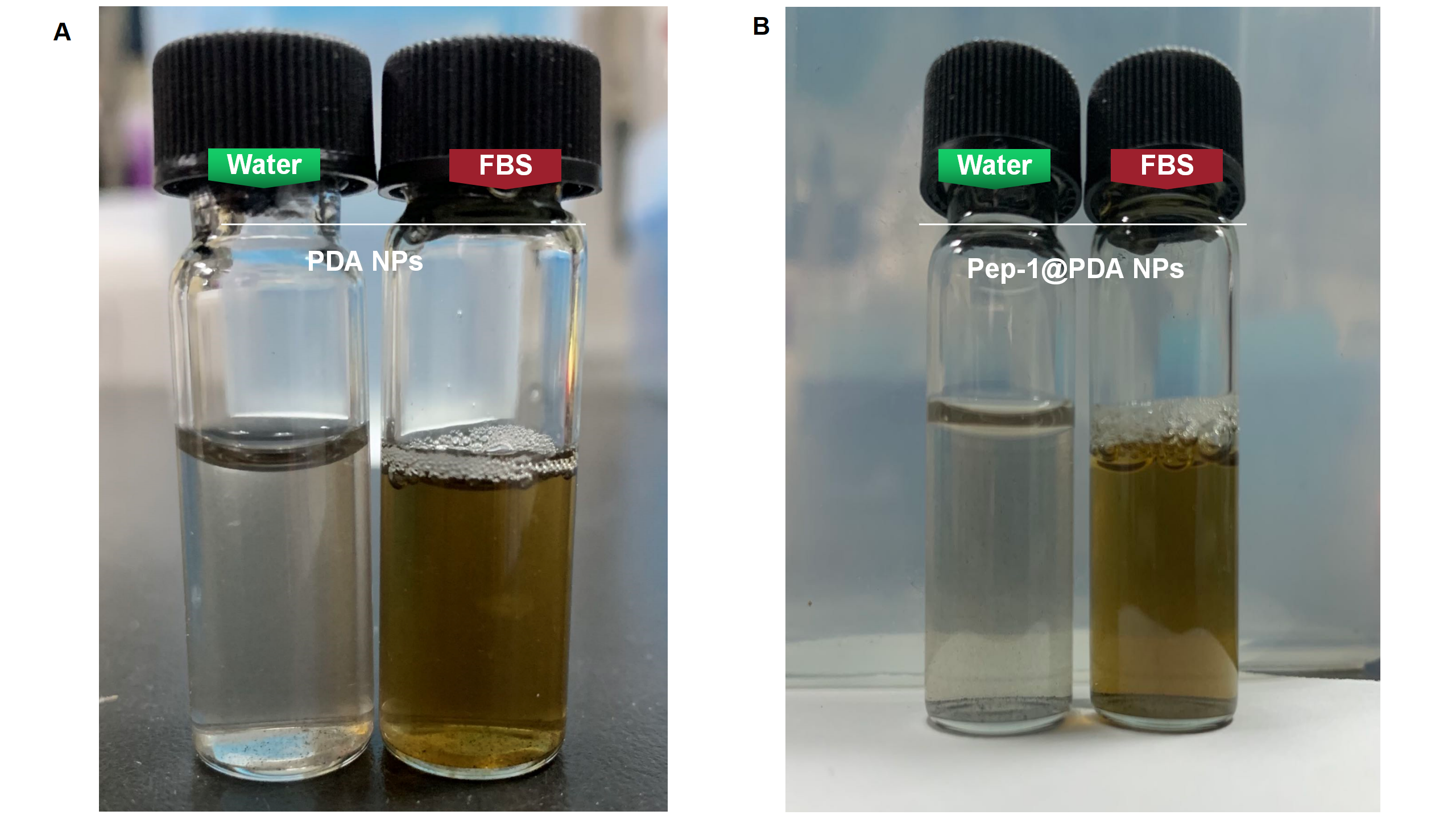

Supplement: Supplementary file 4 [file Image2.TIF]

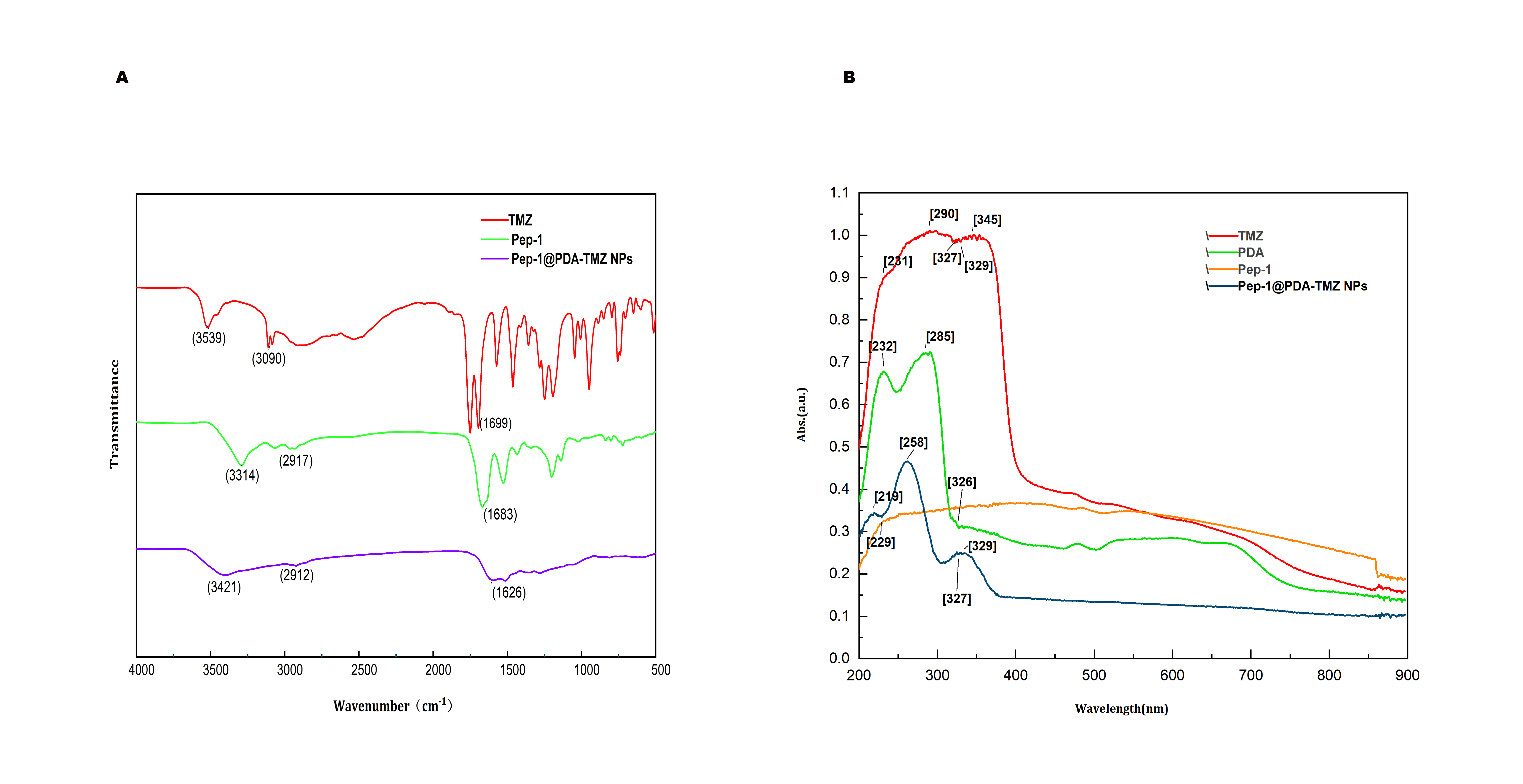

Supplement: Supplementary file 5 [file Image1.TIF]
